# Synthesis and structure of penta­kis­(2-aminopyridinium) nona­vanado(V)tellurate(VI)

**DOI:** 10.1107/S2056989024010533

**Published:** 2024-11-08

**Authors:** Houda Mrad, Ahlem Maalaoui, Mohamed Rzaigui, Samah Akriche

**Affiliations:** a Laboratory of Materials Chemistry (LR13ES08), Faculty of Sciences of Bizerte, University of Carthage, 7021 Zarzouna, Bizerte, Tunisia; University of Aberdeen, United Kingdom

**Keywords:** crystal structure, Hirshfeld surface, polyoxometalate, nona­vanado(V)tellurate(VI), 2-amino­pyridine, non-covalent inter­actions

## Abstract

In the title compound, (C_5_H_7_N_2_)_5_[TeV_9_O_28_], the tellurium and vanadium atoms are statistically disordered over two of the ten metal-atom sites in the [TeV_9_O_28_]^5–^ heteropolyanion. The anions stack along [100] and are extended into a three-dimensional supra­molecular network through N—H⋯O and weak C—H⋯O hydrogen bonds involving the self-assembled 2-amino­pyridinium penta­mers, which are linked by C—H⋯π and π–π stacking inter­actions. The most important contributions to the Hirshfeld surface arise from O⋯H/H⋯O (54.8%), H⋯H (17.8%) and C⋯H/H⋯C (13.4%) contacts.

## Abstract 2

In the title compound, the tellurium(VI) and vanadium(V) atoms are statistically disordered over two of the ten metal-atom sites in the unprotonated [TeV_9_O_28_]^5–^ heteropolyanion.

## 1. Chemical context

Tellurium(VI) often occurs as a central octa­hedral heteroatom in polyoxometalates (POMs) but Te^VI^ is rarely seen in deca­vanadate (V10) structures: just two vanadotellurates with a deca­vanadate structure have been reported, *viz*. the monosubstituted tellurium derivative [H_*x*_TeV_9_O_28_]^(5–*x*)–^ described by Konaka *et al.* (2011 ▸) and the disubstituted species [Te_2_V_9_O_28_]^4–^ reported by our group in the form of its quinolinium salt (Toumi *et al.*, 2013 ▸). Moreover, amino­pyridine derivatives are commonly used as counter-cations in POMs owing to the easy protonation of their N atoms and their high structural stability (Maalaoui *et al.*, 2013 ▸, 2024 ▸; Yuan *et al.*, 2009 ▸). Here we report the synthesis, structure and Hirshfeld surface analyses of the first unprotonated nona­vanado(V)tellurate(VI) cluster, [TeV_9_O_28_]^5–^, crystallized as its anhydrous 2-amino­pyridinium salt, (C_5_H_7_N_2_)_5_[TeV_9_O_28_], (I).
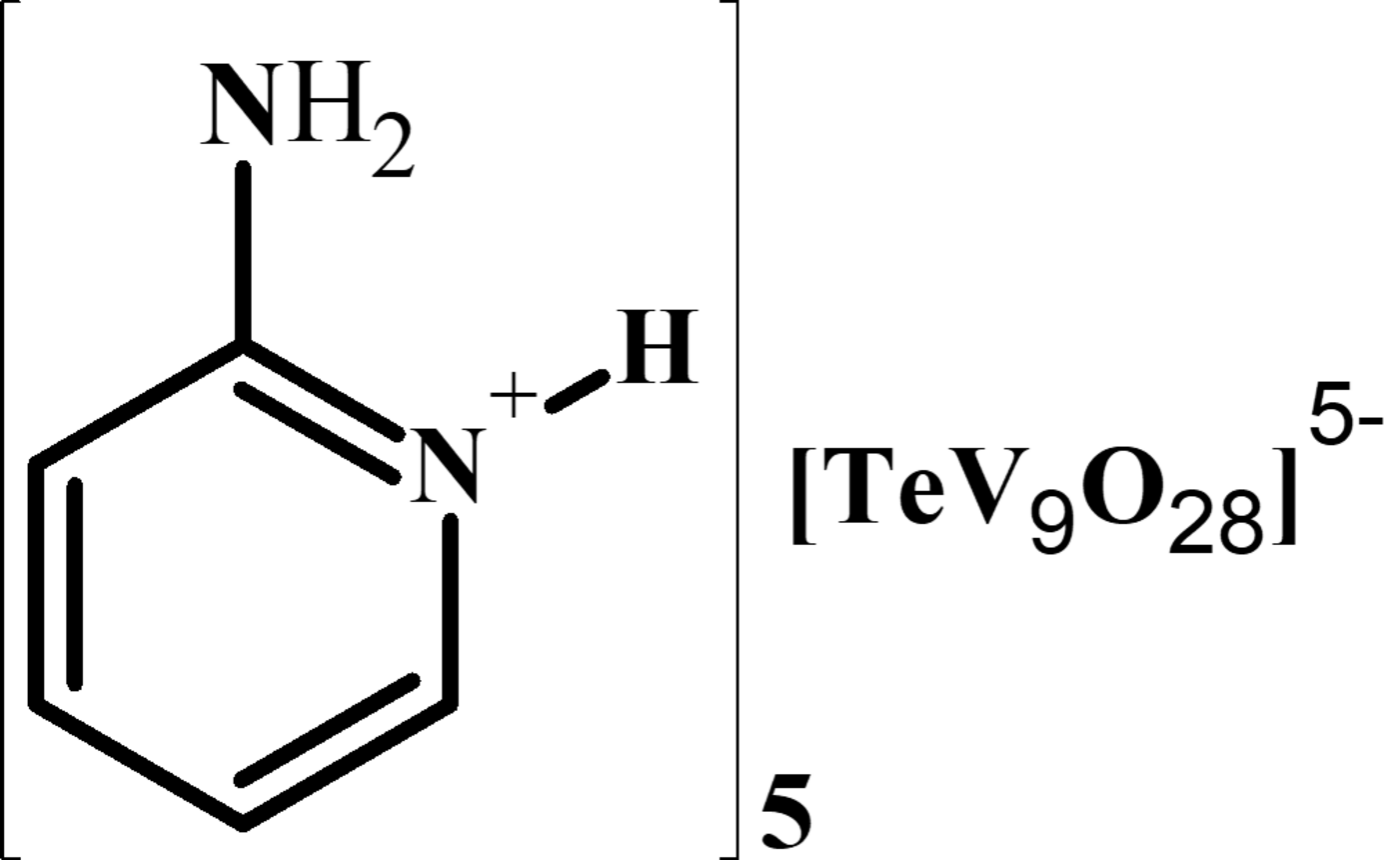


## 2. Structural commentary

**Figure 1:**
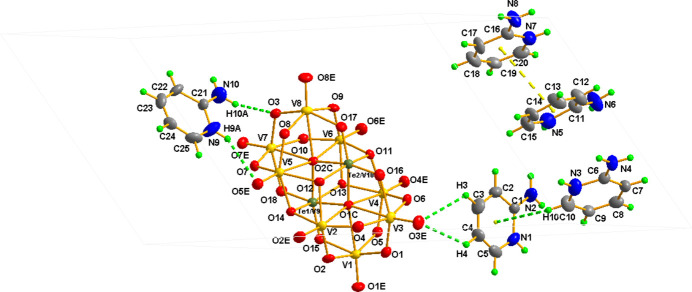
The mol­ecular structure of (I) with displacement ellipsoids drawn at the 50% probability level. H atoms are presented as small spheres of arbitrary radius. Non-covalent inter­molecular inter­actions are shown as dotted lines.

The asymmetric unit of (I) consists of one unprotonated [TeV_9_O_28_]^5–^ heteropolyanion and five 2-amino­pyridinium counter-cations as depicted in Fig. 1 ▸. The structure of the heteropolyanion in (I) belongs to the deca­vanadate structure type (Lee, 2006 ▸), but with tellurium replacing one of the vanadium atoms. The Te heteroatom is statistically distributed over the Te1/V9 and Te2/V10 sites in the title compound. This observation is consistent with the structures reported by Konaka *et al.* (2011 ▸) in TBA_3_[H_2_TeV_9_O_28_] and TBA_4_[HTeV_9_O_28_]·2CH_3_CN (TBA = tetra­butyl ammonium). In the [TeV_9_O_28_]^5–^ polyanion in (I), the VO_6_ octa­hedra are significantly distorted [range of V—O bond lengths = 1.595 (4)– 2.429 (4) Å] whereas the TeO_6_ substituted octa­hedra [Te/V—O ranges 1.769 (4)–2.063 (4) Å] are less distorted in comparison with the VO_6_ octa­hedra. The bond-valence sums (BVS; Brown & Altermatt, 1985 ▸) for Te1 and Te2 are +6.33 and +6.39 v.u. (v.u. = valence units), whereas those for the V cations are in the range +5.17 v.u. to +5.26 v.u., which are consistent with the oxidation states of Te (+VI) and V (+V). An examination of the 2–amino­pyridinium cations show that the bond distances and angles are in accordance with those in analogous salts such as (C_5_H_7_N_2_)_6_[V_10_O_28_]·2H_2_O, (Yuan *et al.*, 2009 ▸), (C_5_H_7_N_2_)_2_ [ReVW_4_O_19_]·7H_2_O (Maaloui *et al.*, 2013 ▸) and (C_5_H_7_N_2_)_5_[PV_2_W_10_O_40_]·0.5(C_5_H_5_N)·2H_2_O (Maaloui *et al.*, 2024 ▸).

## 3. Supra­molecular features

**Figure 2:**
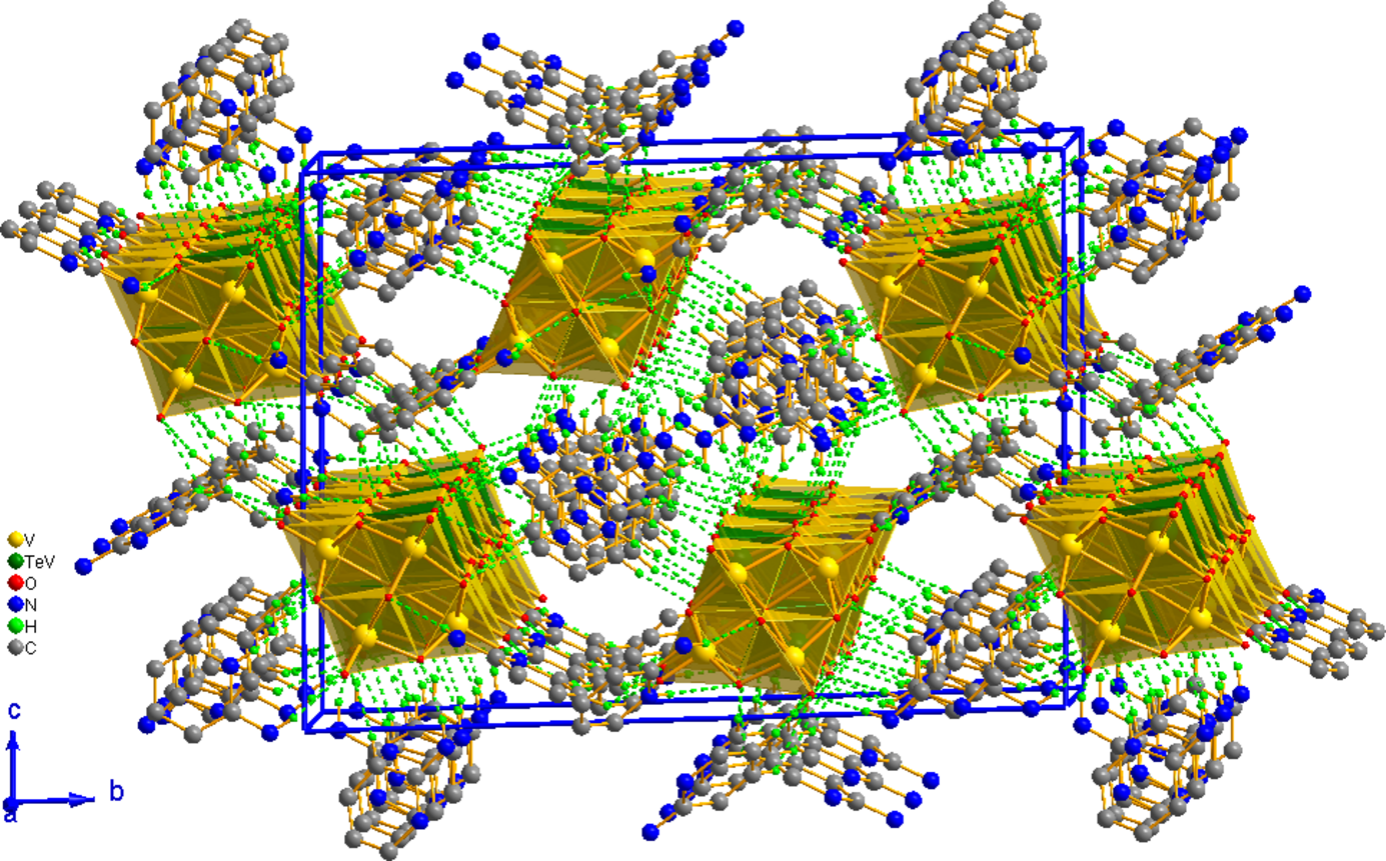
The crystal packing of (I) with N—H⋯O and weak C—H⋯O inter­actions forming a three-dimensional supra­molecular network. H atoms not involved in hydrogen bonding scheme are omitted

**Figure 3:**
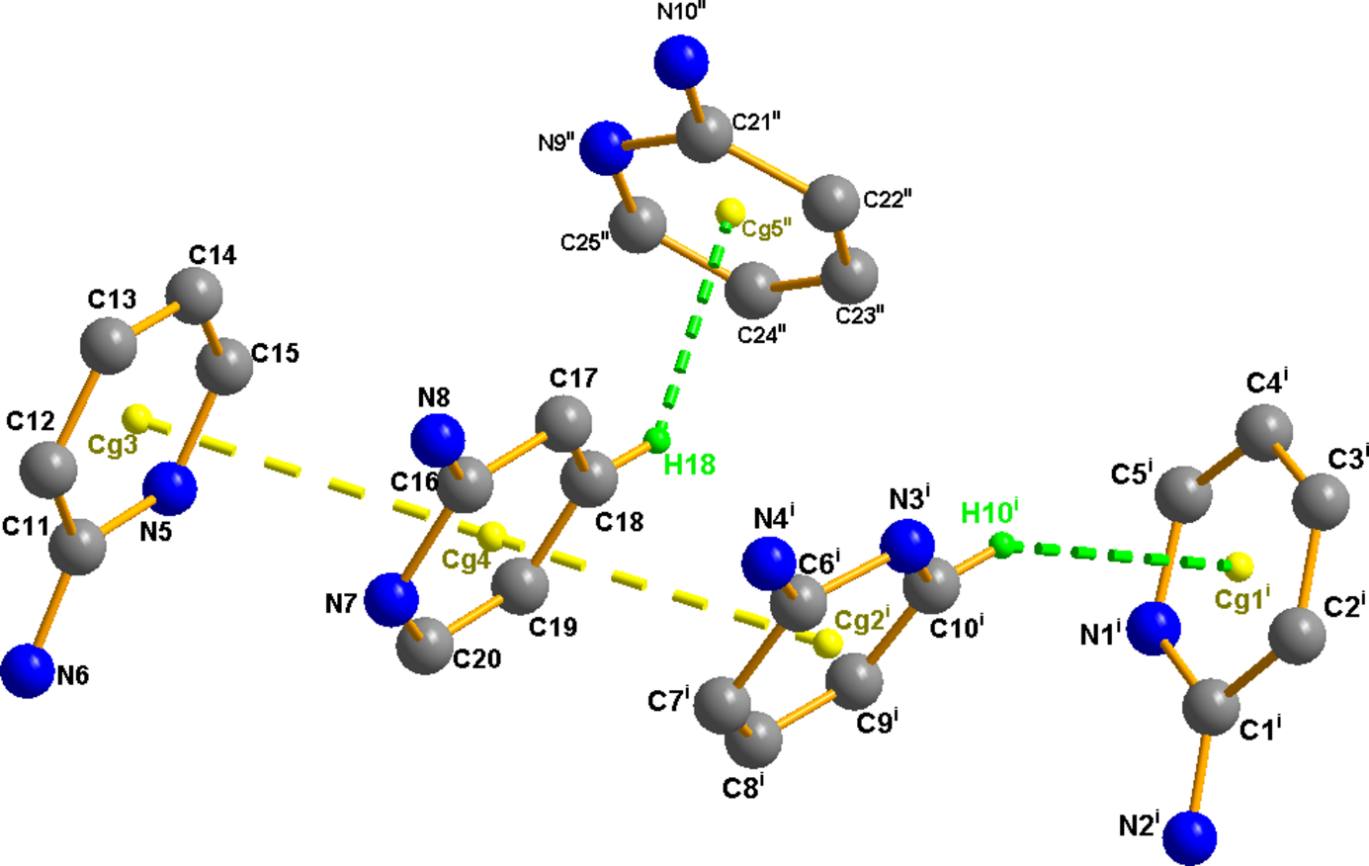
The self-assembled 2-amino­pyridinium penta­mer featuring weak C—H⋯π and π–π stacking inter­actions (depicted by dashed lines). Other H atoms omitted. Symmetry codes: (iv): *x*, 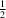 − *y*, 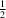 + *z*; (viii): 1 + *x*, *y*, *z*.

In the extended structure, all the hydrogen-bond donors are provided by the five 2-amino­pyridinium cations as the polyanion is unprotonated. Each cation donates hydrogen bonds to the terminal and bridging O atoms of the polyanions stacked along [100] at *z* = 1/4 and 3/4 by means of N—H⋯O and weak C—H⋯O inter­actions, giving rise to a three-dimensional supra­molecular network (Fig. 2 ▸ and Table 1 ▸). Furthermore, the 2-amino­pyridinium moieties are themselves connected by weak C—H⋯π [C10—H10⋯*Cg*1 = 3.066 Å, C18—H18⋯*Cg*5^iv^ = 2.659 Å; symmetry code: (iv) *x*, 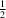 − *y*, 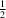 + *z*; Table 1 ▸] and π–π stacking inter­actions between the *R*2/*R*4 and *R*4/*R*3 pyridyl rings [*R*1 = N1/C1–C5 (centroid *Cg*1), *R*2 = N3/C6–C10 (centroid *Cg*2), *R*3 = N5/C11–C15 (centroid *Cg*3), *R*4 = N7/C16–C20 (centroid *Cg*4), R5 = N9/C21–C25 (centroid *Cg*5)] stacked in parallel displaced face-to-face arrangements with centroid–centroid distances of 3.724 (2) and 3.829 (2) Å, respectively (Fig. 3 ▸), within the accepted rangesfor C—H⋯π and π–π stacking interactions (Janiak (2000 ▸).

## 4. Hirshfeld surface analysis

**Figure 4:**
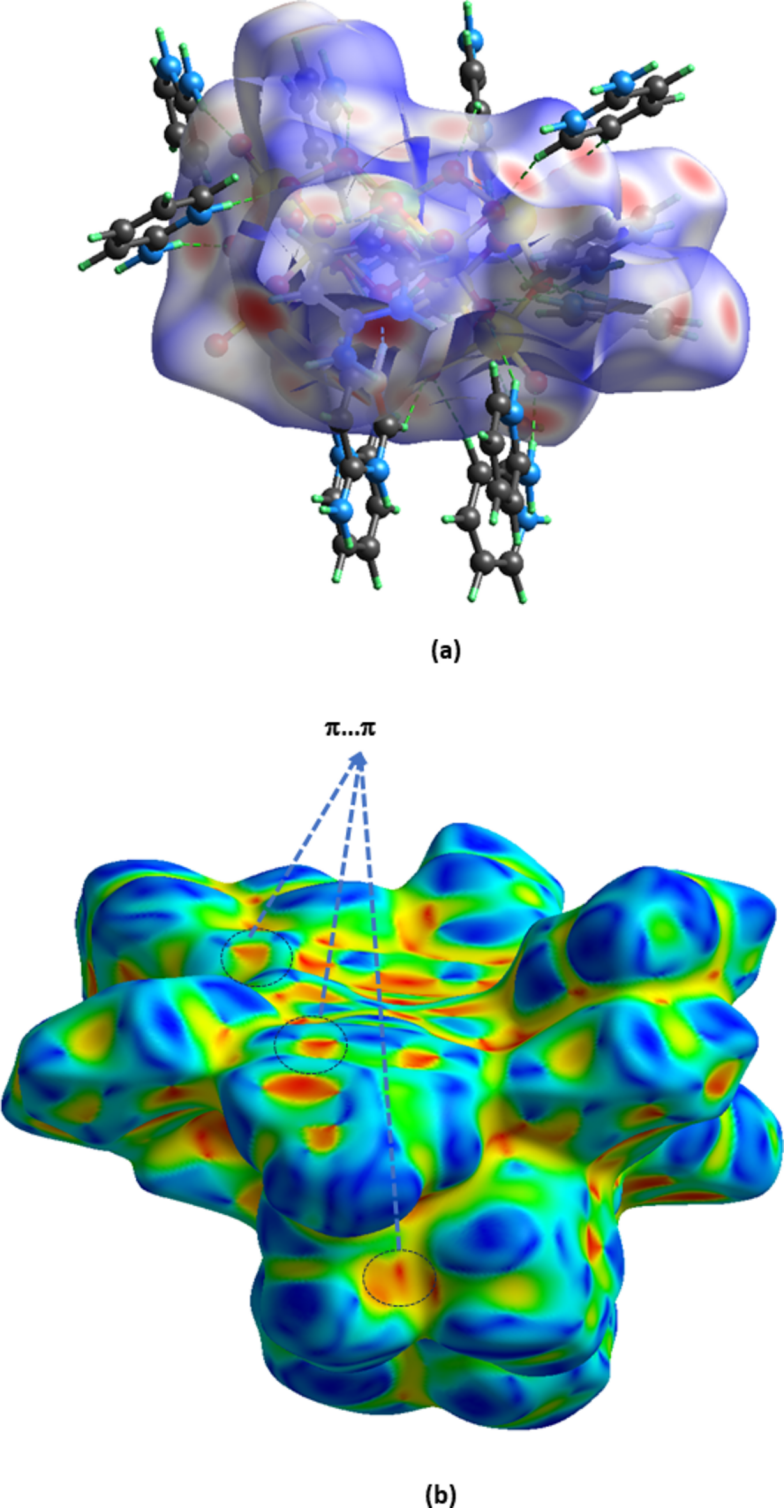
The Hirshfeld surface mapped over (*a*) *d*_norm_ and (*b*) shape-index. N—H⋯O and C—H⋯O hydrogen bonds from neighbouring organic cations are represented by green dotted lines.

**Figure 5:**
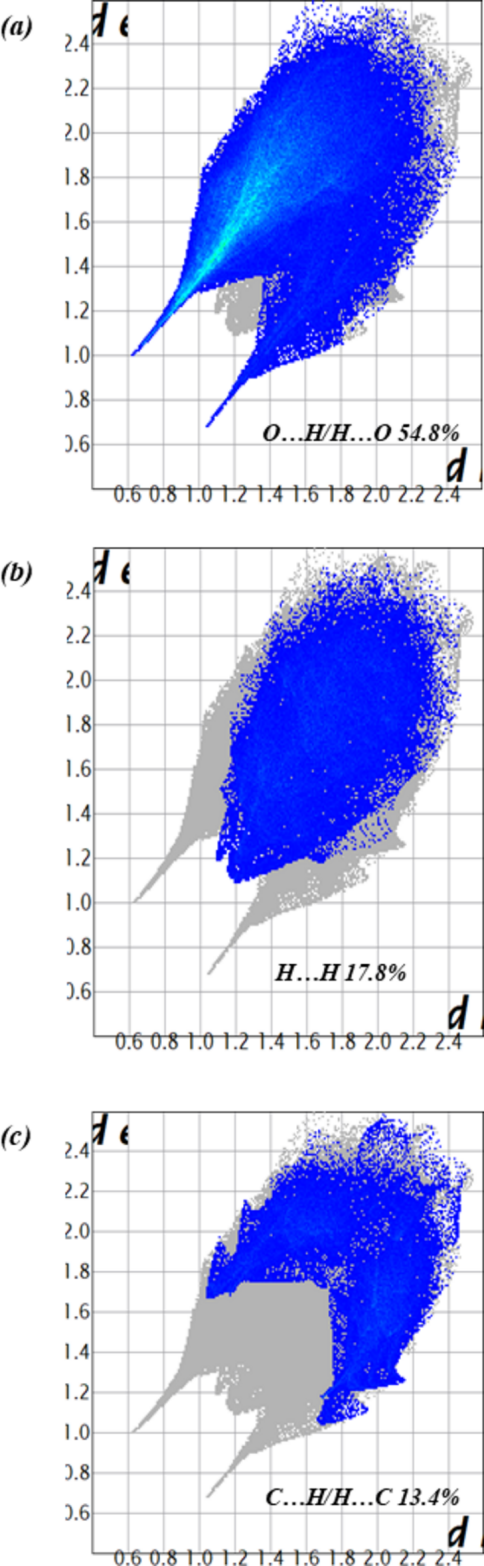
Fingerprint plots showing the major contacts contributions of (*a*) H⋯O/O⋯H, (*b*) H⋯H and (*c*) C⋯H/H⋯C.

Fig. 4 ▸(*a*) illustrates the Hirshfeld surface of (I) mapped over *d*_norm_ with red spots corresponding to short inter-contacts. The red, triangular concave regions in the Hirshfeld surface mapped with shape index [Fig. 4 ▸(*b*)], confirm the existence of the π–π stacking inter­actions mentioned above. The fingerprint plots (Fig. 5 ▸) indicate that the major contact contributions to the crystal structure are from O⋯H/H⋯O (54.8%), H⋯H (17.8%) and C⋯H/H⋯C (13.4%) whereas the contributions of the remaining contacts [N⋯H/H⋯N (4.7%), O⋯C/C⋯O (2.6%), C⋯C (2.3%), O⋯O (1.6%)] are very small. The characteristic spikes in the O⋯H/H⋯O plot [Fig. 5 ▸(*a*)] indicate the existence of the N—H⋯O and C—H⋯O hydrogen bonds (Table 1 ▸).

## 5. Database survey

Related vanadotellurates with the deca­vanadate structure type include TBA_4_[HTeV_9_O_28_]·2CH_3_CN and TBA_3_[H_2_TeV_9_O_28_] (TBA = tetra-*n*-butyl­ammonium) (Konaka *et al.*, 2011 ▸) and (C_9_H_8_N)_4_[Te_2_V_8_O_28_]·8H_2_O (Toumi *et al.*, 2013 ▸). Related nona­vanadoplatinate(IV) clusters include Na_9_[H_2_PtV_9_O_28_][H_3_PtV_9_O_28_]·40H_2_O (Joo *et al.*, 2015 ▸), Na_5_[H_2_PtV_9_O_28_]·21H_2_O (Lee *et al.*, 2008 ▸), (CH_6_N_3_)_5_[H_2_PtV_9_O_28_] (Joo *et al.*, 2011 ▸) and K_5_[H_2_PtV_9_O_28_]·9H_2_O (Joo & Lee, 2015 ▸). For the related structures of amino­pyridinium containing polyoxometalates (C_5_H_7_N_2_)_6_[V_10_O_28_]·2H_2_O, see: Yuan *et al.* (2009 ▸); (C_5_H_7_N_2_)_2_[ReVW_4_O_19_]·7H_2_O, see: Maaloui *et al.* (2013 ▸) and (C_5_H_7_N_2_)_5_[PV_2_W_10_O_40_]·0.5(C_5_H_5_N)·2H_2_O, see: Maaloui *et al.* (2024 ▸).

## 6. Synthesis and crystallization

Vanadium(V) oxide (1.26 g, 6.93 mmol), 2-amino­pyridine (0.59 g, 6.16 mmol) and telluric acid, Te(OH)_6_ (0.36 g, 1.55 mmol) were suspended in 50 ml of distilled water. Then the pH value of the mixture was adjusted to 6 with 3 *M* hydro­chloric acid (HCl) and stirred for 3 h. After one week, yellow prismatic single crystals were grown by slow evaporation at room temperature.

## 7. Refinement

Crystal data, data collection and structure refinement details are summarized in Table 2 ▸. All H-atoms were positioned with idealized geometry and refined using a riding model with C—H = 0.93 Å and *U*_iso_(H) = 1.2*U*_eq_(C) and N—H = 0.86 Å and *U*_iso_(H) = 1.2*U*_eq_(N).

## Supplementary Material

Crystal structure: contains datablock(s) I. DOI: 10.1107/S2056989024010533/hb8109sup1.cif

Structure factors: contains datablock(s) I. DOI: 10.1107/S2056989024010533/hb8109Isup3.hkl

CCDC reference: 2333283

Additional supporting information:  crystallographic information; 3D view; checkCIF report

## Figures and Tables

**Table 1:** Hydrogen-bond geometry (Å, °)

*D*—H⋯*A*	*D*—H	H⋯*A*	*D*⋯*A*	*D*—H⋯*A*
N1—H1⋯O6^i^	0.86	2.57	3.426 (8)	172
N2—H2*A*⋯O1^i^	0.86	2.26	3.105 (8)	167
N2—H2*B*⋯O3^ii^	0.86	2.50	3.006 (7)	118
N2—H2*B*⋯O10^ii^	0.86	2.28	3.121 (8)	165
C4—H4⋯O3*E*	0.93	2.44	2.995 (8)	119
C4—H4⋯O8*E*^iii^	0.93	2.26	3.073 (8)	146
C5—H5⋯O9^iii^	0.93	2.24	3.136 (8)	161
N3—H3*A*⋯O12^iv^	0.86	2.54	3.397 (7)	175
N4—H4*A*⋯O7^v^	0.86	2.05	2.896 (7)	168
N4—H4*B*⋯O16^iv^	0.86	2.19	2.998 (7)	156
C7—H7⋯O18^v^	0.93	2.57	3.495 (8)	171
C9—H9⋯O5^i^	0.93	1.86	2.760 (7)	161
N5—H5*A*⋯O10^ii^	0.86	2.61	3.404 (8)	154
N6—H6*A*⋯O1*E*^vi^	0.86	2.14	2.922 (8)	151
N6—H6*B*⋯O7*E*^ii^	0.86	2.05	2.900 (8)	171
C12—H12⋯O1*E*^vi^	0.93	2.63	3.312 (9)	130
C14—H14⋯O8^iv^	0.93	1.78	2.703 (7)	174
C14—H14⋯O17^iv^	0.93	2.62	3.146 (8)	117
N7—H7*A*⋯O15^vi^	0.86	2.48	3.308 (8)	161
N8—H8*A*⋯O3*E*^vii^	0.86	2.12	2.966 (7)	169
N8—H8*B*⋯O14^vi^	0.86	2.18	2.963 (7)	152
C19—H19⋯O13^ii^	0.93	1.88	2.788 (7)	165
C20—H20⋯O18^ii^	0.93	2.43	3.202 (8)	140
N9—H9*A*⋯O5*E*	0.86	2.46	3.244 (7)	151
N10—H10*A*⋯O3	0.86	2.30	3.098 (8)	155
N10—H10*B*⋯O1^viii^	0.86	2.65	3.294 (8)	132
N10—H10*B*⋯O2^viii^	0.86	2.34	3.169 (8)	162
C22—H22⋯O2*E*^viii^	0.93	2.29	3.189 (8)	164
C24—H24⋯O4*E*^ix^	0.93	2.35	2.929 (7)	120
C24—H24⋯O6*E*^ix^	0.93	2.61	3.153 (7)	118
C24—H24⋯O11^x^	0.93	2.48	3.003 (7)	116
C10—H10⋯*Cg*1	0.93	3.07	3.902	151
C18—H18⋯*Cg*5^iv^	0.93	2.66	3.450	143

Symmetry codes: (i) 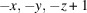; (ii) 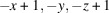; (iii) 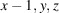; (iv) 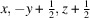; (v) 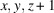; (vi) 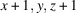; (vii) 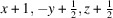; (viii) 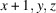; (ix) 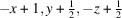; (x) 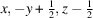.

**Table 2:** Experimental details

Crystal data	
Chemical formula	(C_5_H_7_N_2_)_5_[TeV_9_O_28_]
*M*_r_	1509.69
Crystal system, space group	Monoclinic, *P*2_1_/*c*
Temperature (K)	293
*a*, *b*, *c* (Å)	11.661 (2), 23.251 (2), 19.602 (3)
β (°)	122.53 (1)
*V* (Å^3^)	4481.1 (12)
*Z*	4
Radiation type	Ag *K*α, λ = 0.56087 Å
μ (mm^−1^)	1.31
Crystal size (mm)	0.25 × 0.19 × 0.13

Data collection	
Diffractometer	Enraf–Nonius CAD-4
Absorption correction	Multi-scan (Blessing, 1995 ▸)
*T*_min_, *T*_max_	0.745, 0.807
No. of measured, independent and observed [*I* > 2σ(*I*)] reflections	25752, 21889, 10265
*R*_int_	0.048
(sin θ/λ)_max_ (Å^−1^)	0.837

Refinement	
*R*[*F*^2^ > 2σ(*F*^2^)], *wR*(*F*^2^), *S*	0.076, 0.211, 1.03
No. of reflections	21889
No. of parameters	658
H-atom treatment	H-atom parameters constrained
Δρ_max_, Δρ_min_ (e Å^−3^)	1.19, −1.74

Computer programs: *CAD-4 EXPRESS* (Enraf–Nonius, 1994 ▸), *XCAD4* (Harms & Wocadlo, 1996 ▸), *SHELXS97* (Sheldrick, 2008 ▸), *SHELXL2018/3* (Sheldrick, 2015 ▸), *DIAMOND* (Brandenburg & Putz, 2005 ▸), *WinGX* publication routines (Farrugia, 2012 ▸) and *CrystalExplorer* (Wolff *et al.*, 2012 ▸).

## supplementary crystallographic information

### Pentakis(2-aminopyridinium) nonavanado(V)tellurate(VI) Crystal data

**Table:**

(C_5_H_7_N_2_)_5_[TeV_9_O_28_]	*F*(000) = 2952
*M**_r_* = 1509.69	*D*_x_ = 2.238 Mg m^−^^3^
Monoclinic, *P*2_1_/*c*	Ag *K*α radiation, λ = 0.56087 Å
*a* = 11.661 (2) Å	Cell parameters from 25 reflections
*b* = 23.251 (2) Å	θ = 9–11°
*c* = 19.602 (3) Å	µ = 1.31 mm^−^^1^
β = 122.53 (1)°	*T* = 293 K
*V* = 4481.1 (12) Å^3^	Prism, yellow
*Z* = 4	0.25 × 0.19 × 0.13 mm

### Pentakis(2-aminopyridinium) nonavanado(V)tellurate(VI) Data collection

**Table:**

Enraf–Nonius CAD-4 diffractometer	10265 reflections with *I* > 2σ(*I*)
Radiation source: Enraf Nonius FR590	*R*_int_ = 0.048
Graphite monochromator	θ_max_ = 28.0°, θ_min_ = 2.1°
non–profiled ω scans	*h* = −19→19
Absorption correction: multi-scan (Blessing, 1995)	*k* = −38→1
*T*_min_ = 0.745, *T*_max_ = 0.807	*l* = −21→32
25752 measured reflections	2 standard reflections every 120 min
21889 independent reflections	intensity decay: −2%

### Pentakis(2-aminopyridinium) nonavanado(V)tellurate(VI) Refinement

**Table:**

Refinement on *F*^2^	Primary atom site location: structure-invariant direct methods
Least-squares matrix: full	Secondary atom site location: difference Fourier map
*R*[*F*^2^ > 2σ(*F*^2^)] = 0.076	Hydrogen site location: difference Fourier map
*wR*(*F*^2^) = 0.211	H-atom parameters constrained
*S* = 1.03	*w* = 1/[σ^2^(*F*_o_^2^) + (0.0646*P*)^2^ + 23.768*P*] where *P* = (*F*_o_^2^ + 2*F*_c_^2^)/3
21889 reflections	(Δ/σ)_max_ < 0.001
658 parameters	Δρ_max_ = 1.19 e Å^−^^3^
0 restraints	Δρ_min_ = −1.74 e Å^−^^3^

### Pentakis(2-aminopyridinium) nonavanado(V)tellurate(VI) Special details

**Table:**

Geometry. All esds (except the esd in the dihedral angle between two l.s. planes) are estimated using the full covariance matrix. The cell esds are taken into account individually in the estimation of esds in distances, angles and torsion angles; correlations between esds in cell parameters are only used when they are defined by crystal symmetry. An approximate (isotropic) treatment of cell esds is used for estimating esds involving l.s. planes.

### Pentakis(2-aminopyridinium) nonavanado(V)tellurate(VI) Fractional atomic coordinates and isotropic or equivalent isotropic displacement parameters (Å^2^)

**Table:**

	*x*	*y*	*z*	*U*_iso_*/*U*_eq_	Occ. (<1)
V1	−0.04965 (9)	0.09075 (4)	0.20903 (6)	0.02450 (19)	
V2	0.08300 (10)	0.20810 (4)	0.20870 (6)	0.02400 (19)	
V3	0.10819 (10)	0.16241 (4)	0.36755 (6)	0.02479 (19)	
V4	0.22827 (9)	0.04297 (4)	0.35456 (6)	0.02207 (18)	
V5	0.33925 (9)	0.20776 (4)	0.19507 (6)	0.02098 (18)	
V6	0.48299 (9)	0.04162 (4)	0.33892 (6)	0.02189 (18)	
V7	0.45911 (10)	0.09044 (4)	0.18122 (6)	0.02306 (18)	
V8	0.61987 (9)	0.15934 (4)	0.34255 (6)	0.02428 (19)	
Te1	0.20032 (5)	0.08920 (2)	0.19110 (3)	0.02224 (11)	0.5
V9	0.20032 (5)	0.08920 (2)	0.19110 (3)	0.02224 (11)	0.5
Te2	0.36502 (5)	0.16117 (2)	0.35539 (3)	0.01968 (10)	0.5
V10	0.36502 (5)	0.16117 (2)	0.35539 (3)	0.01968 (10)	0.5
O1C	0.1777 (4)	0.12502 (15)	0.2795 (2)	0.0194 (7)	
O2C	0.3881 (4)	0.12507 (15)	0.2679 (2)	0.0184 (7)	
O1E	−0.1978 (4)	0.0632 (2)	0.1558 (3)	0.0345 (10)	
O2E	0.0345 (4)	0.26655 (19)	0.1575 (3)	0.0334 (10)	
O3E	0.0777 (5)	0.18882 (19)	0.4322 (3)	0.0349 (10)	
O4E	0.2796 (4)	−0.01547 (18)	0.4051 (3)	0.0291 (9)	
O5E	0.2880 (4)	0.26650 (18)	0.1446 (3)	0.0303 (9)	
O6E	0.5314 (4)	−0.01715 (18)	0.3887 (3)	0.0323 (9)	
O7E	0.4904 (5)	0.0649 (2)	0.1165 (3)	0.0331 (10)	
O8E	0.7705 (4)	0.1847 (2)	0.3991 (3)	0.0351 (10)	
O1	−0.0473 (4)	0.12517 (18)	0.2928 (3)	0.0279 (8)	
O2	−0.0658 (4)	0.16167 (18)	0.1600 (3)	0.0281 (8)	
O3	0.6150 (4)	0.12598 (17)	0.2562 (2)	0.0253 (8)	
O4	0.0704 (4)	0.22175 (17)	0.2957 (3)	0.0274 (8)	
O5	0.0520 (4)	0.02864 (17)	0.2764 (2)	0.0252 (8)	
O6	0.1898 (4)	0.09013 (18)	0.4131 (2)	0.0254 (8)	
O7	0.3775 (4)	0.16162 (17)	0.1356 (2)	0.0238 (8)	
O8	0.5151 (4)	0.22191 (17)	0.2729 (2)	0.0241 (8)	
O9	0.6298 (4)	0.08877 (17)	0.3882 (2)	0.0250 (8)	
O10	0.4971 (4)	0.02920 (17)	0.2523 (2)	0.0242 (8)	
O11	0.4076 (4)	0.08450 (16)	0.3978 (2)	0.0201 (7)	
O12	0.2858 (4)	0.22228 (15)	0.2773 (2)	0.0207 (7)	
O13	0.2804 (4)	0.02707 (16)	0.2701 (2)	0.0196 (7)	
O14	0.1581 (4)	0.16667 (16)	0.1492 (2)	0.0218 (7)	
O15	0.0311 (4)	0.06384 (17)	0.1429 (2)	0.0250 (8)	
O16	0.3056 (4)	0.18699 (17)	0.4170 (2)	0.0258 (8)	
O17	0.5353 (4)	0.18601 (17)	0.4046 (2)	0.0244 (8)	
O18	0.2607 (4)	0.06431 (17)	0.1304 (2)	0.0259 (8)	
N1	−0.0124 (6)	0.0239 (3)	0.5776 (4)	0.0454 (15)	
H1	−0.0643	−0.0021	0.5783	0.055*	
N2	0.1895 (7)	−0.0268 (3)	0.6727 (4)	0.0466 (16)	
H2A	0.1414	−0.0544	0.6739	0.056*	
H2B	0.2768	−0.0288	0.7019	0.056*	
C1	0.1300 (6)	0.0180 (3)	0.6268 (4)	0.0304 (12)	
C2	0.2068 (7)	0.0636 (3)	0.6223 (4)	0.0385 (15)	
H2	0.3013	0.0623	0.6537	0.046*	
C3	0.1435 (9)	0.1084 (3)	0.5729 (5)	0.0475 (18)	
H3	0.1952	0.1377	0.5701	0.057*	
C4	0.0094 (7)	0.1119 (3)	0.5281 (4)	0.0350 (14)	
H4	−0.0318	0.1438	0.4950	0.042*	
C5	−0.0669 (7)	0.0704 (4)	0.5297 (4)	0.0445 (18)	
H5	−0.1611	0.0735	0.4968	0.053*	
N3	0.2068 (7)	0.1563 (3)	0.8399 (4)	0.0481 (16)	
H3A	0.2313	0.1862	0.8249	0.058*	
N4	0.3077 (6)	0.1934 (2)	0.9747 (3)	0.0378 (13)	
H4A	0.3277	0.1892	1.0236	0.045*	
H4B	0.3303	0.2243	0.9609	0.045*	
C6	0.2417 (6)	0.1519 (3)	0.9207 (4)	0.0293 (12)	
C7	0.2030 (7)	0.1005 (3)	0.9413 (4)	0.0332 (13)	
H7	0.2255	0.0945	0.9940	0.040*	
C8	0.1334 (7)	0.0602 (3)	0.8843 (5)	0.0390 (15)	
H8	0.1085	0.0266	0.8987	0.047*	
C9	0.0989 (6)	0.0666 (2)	0.8084 (4)	0.0284 (11)	
H9	0.0495	0.0379	0.7708	0.034*	
C10	0.1343 (8)	0.1138 (3)	0.7853 (5)	0.0451 (17)	
H10	0.1096	0.1177	0.7318	0.054*	
N5	0.5217 (7)	0.0852 (3)	0.8616 (4)	0.0536 (17)	
H5A	0.4945	0.0536	0.8348	0.064*	
N6	0.6012 (8)	0.0360 (3)	0.9866 (4)	0.057 (2)	
H6A	0.6405	0.0363	1.0384	0.068*	
H6B	0.5752	0.0040	0.9606	0.068*	
C11	0.5809 (7)	0.0847 (3)	0.9469 (4)	0.0351 (14)	
C12	0.6208 (8)	0.1373 (3)	0.9858 (5)	0.0483 (19)	
H12	0.6587	0.1394	1.0413	0.058*	
C13	0.6045 (9)	0.1859 (3)	0.9429 (5)	0.054 (2)	
H13	0.6326	0.2210	0.9697	0.065*	
C14	0.5497 (7)	0.1844 (3)	0.8637 (4)	0.0382 (15)	
H14	0.5397	0.2183	0.8358	0.046*	
C15	0.5084 (8)	0.1344 (3)	0.8233 (5)	0.0474 (18)	
H15	0.4699	0.1341	0.7677	0.057*	
N7	0.9043 (7)	0.1101 (3)	0.9559 (4)	0.0486 (16)	
H7A	0.9288	0.1059	1.0056	0.058*	
N8	0.9909 (7)	0.2065 (3)	0.9791 (4)	0.0481 (16)	
H8A	1.0041	0.2383	0.9620	0.058*	
H8B	1.0190	0.2025	1.0294	0.058*	
C16	0.9275 (7)	0.1637 (3)	0.9281 (4)	0.0342 (13)	
C17	0.8790 (8)	0.1676 (3)	0.8452 (4)	0.0428 (17)	
H17	0.8876	0.2017	0.8236	0.051*	
C18	0.8194 (8)	0.1207 (3)	0.7970 (4)	0.0479 (19)	
H18	0.7905	0.1229	0.7427	0.057*	
C19	0.8012 (7)	0.0718 (3)	0.8253 (4)	0.0350 (14)	
H19	0.7582	0.0410	0.7902	0.042*	
C20	0.8432 (7)	0.0662 (3)	0.9024 (5)	0.0415 (16)	
H20	0.8306	0.0313	0.9206	0.050*	
N9	0.5489 (7)	0.2953 (3)	0.1326 (5)	0.058 (2)	
H9A	0.5021	0.2796	0.1497	0.070*	
N10	0.7082 (7)	0.2180 (3)	0.1817 (4)	0.0533 (17)	
H10A	0.6639	0.2009	0.1995	0.064*	
H10B	0.7802	0.2024	0.1880	0.064*	
C21	0.6657 (6)	0.2680 (3)	0.1446 (4)	0.0305 (12)	
C22	0.7364 (7)	0.2980 (3)	0.1143 (5)	0.0393 (15)	
H22	0.8148	0.2823	0.1203	0.047*	
C23	0.6885 (8)	0.3491 (3)	0.0767 (4)	0.0427 (17)	
H23	0.7332	0.3673	0.0552	0.051*	
C24	0.5814 (6)	0.3742 (2)	0.0694 (4)	0.0313 (13)	
H24	0.5564	0.4109	0.0474	0.038*	
C25	0.5100 (7)	0.3475 (3)	0.0933 (5)	0.0457 (18)	
H25	0.4301	0.3645	0.0832	0.055*	

### Pentakis(2-aminopyridinium) nonavanado(V)tellurate(VI) Atomic displacement parameters (Å^2^)

**Table:**

	*U*^11^	*U*^22^	*U*^33^	*U*^12^	*U*^13^	*U*^23^
V1	0.0177 (4)	0.0258 (5)	0.0284 (5)	−0.0012 (4)	0.0114 (4)	0.0006 (4)
V2	0.0217 (4)	0.0225 (4)	0.0293 (5)	0.0046 (3)	0.0147 (4)	0.0060 (4)
V3	0.0289 (5)	0.0225 (4)	0.0302 (5)	0.0015 (4)	0.0207 (4)	−0.0011 (4)
V4	0.0231 (4)	0.0190 (4)	0.0253 (4)	0.0007 (3)	0.0138 (4)	0.0040 (3)
V5	0.0199 (4)	0.0195 (4)	0.0237 (4)	−0.0004 (3)	0.0119 (4)	0.0035 (3)
V6	0.0195 (4)	0.0201 (4)	0.0254 (4)	0.0027 (3)	0.0116 (4)	0.0030 (3)
V7	0.0223 (4)	0.0253 (4)	0.0239 (4)	0.0002 (4)	0.0141 (4)	−0.0024 (4)
V8	0.0174 (4)	0.0250 (5)	0.0267 (5)	−0.0020 (3)	0.0094 (4)	−0.0028 (4)
Te1	0.0202 (2)	0.0230 (2)	0.0224 (2)	−0.00086 (19)	0.0107 (2)	−0.0001 (2)
V9	0.0202 (2)	0.0230 (2)	0.0224 (2)	−0.00086 (19)	0.0107 (2)	−0.0001 (2)
Te2	0.0208 (2)	0.0180 (2)	0.0200 (2)	0.00005 (18)	0.01080 (18)	0.00028 (18)
V10	0.0208 (2)	0.0180 (2)	0.0200 (2)	0.00005 (18)	0.01080 (18)	0.00028 (18)
O1C	0.0188 (16)	0.0179 (16)	0.0239 (17)	−0.0015 (13)	0.0131 (14)	−0.0024 (14)
O2C	0.0186 (16)	0.0144 (15)	0.0226 (17)	−0.0016 (13)	0.0114 (14)	−0.0013 (13)
O1E	0.0215 (19)	0.042 (3)	0.035 (2)	−0.0065 (18)	0.0117 (18)	−0.002 (2)
O2E	0.029 (2)	0.029 (2)	0.044 (3)	0.0088 (17)	0.021 (2)	0.0153 (19)
O3E	0.047 (3)	0.032 (2)	0.038 (2)	0.001 (2)	0.031 (2)	−0.0063 (19)
O4E	0.032 (2)	0.026 (2)	0.030 (2)	0.0061 (17)	0.0166 (18)	0.0089 (17)
O5E	0.030 (2)	0.029 (2)	0.034 (2)	0.0015 (17)	0.0183 (18)	0.0066 (18)
O6E	0.032 (2)	0.027 (2)	0.038 (2)	0.0079 (18)	0.019 (2)	0.0105 (18)
O7E	0.033 (2)	0.042 (3)	0.031 (2)	−0.0019 (19)	0.0212 (19)	−0.0062 (19)
O8E	0.023 (2)	0.037 (2)	0.037 (2)	−0.0078 (18)	0.0104 (18)	−0.008 (2)
O1	0.028 (2)	0.029 (2)	0.034 (2)	0.0017 (17)	0.0212 (18)	0.0028 (17)
O2	0.0166 (17)	0.028 (2)	0.037 (2)	0.0033 (15)	0.0122 (16)	0.0063 (18)
O3	0.0210 (18)	0.0259 (19)	0.030 (2)	0.0014 (15)	0.0147 (16)	0.0015 (16)
O4	0.029 (2)	0.0196 (18)	0.037 (2)	0.0035 (15)	0.0198 (19)	0.0025 (16)
O5	0.0261 (19)	0.0220 (18)	0.0282 (19)	−0.0037 (15)	0.0150 (16)	0.0015 (15)
O6	0.030 (2)	0.0274 (19)	0.0267 (19)	0.0042 (16)	0.0204 (17)	0.0027 (16)
O7	0.0254 (18)	0.0252 (19)	0.0233 (18)	0.0035 (15)	0.0147 (16)	0.0041 (15)
O8	0.0235 (18)	0.0240 (18)	0.0255 (19)	−0.0027 (15)	0.0136 (16)	−0.0021 (15)
O9	0.0157 (16)	0.0276 (19)	0.0241 (18)	−0.0007 (15)	0.0058 (14)	−0.0019 (16)
O10	0.0233 (18)	0.0223 (18)	0.0277 (19)	0.0027 (14)	0.0141 (16)	−0.0004 (15)
O11	0.0218 (17)	0.0216 (17)	0.0166 (15)	0.0026 (14)	0.0102 (14)	0.0004 (13)
O12	0.0182 (16)	0.0186 (16)	0.0233 (17)	0.0029 (13)	0.0099 (14)	0.0012 (14)
O13	0.0200 (16)	0.0220 (17)	0.0162 (15)	0.0041 (14)	0.0093 (14)	0.0017 (13)
O14	0.0184 (17)	0.0242 (18)	0.0216 (17)	−0.0004 (14)	0.0100 (15)	0.0019 (14)
O15	0.0243 (19)	0.0237 (19)	0.0263 (19)	−0.0024 (15)	0.0132 (16)	−0.0021 (15)
O16	0.031 (2)	0.0213 (18)	0.027 (2)	0.0015 (16)	0.0171 (17)	−0.0018 (15)
O17	0.0229 (18)	0.0236 (19)	0.0225 (18)	−0.0043 (15)	0.0094 (15)	−0.0048 (15)
O18	0.0260 (19)	0.0245 (19)	0.027 (2)	−0.0025 (15)	0.0145 (17)	−0.0060 (16)
N1	0.039 (3)	0.058 (4)	0.052 (4)	−0.007 (3)	0.033 (3)	−0.003 (3)
N2	0.045 (3)	0.047 (4)	0.056 (4)	0.017 (3)	0.033 (3)	0.026 (3)
C1	0.027 (3)	0.038 (3)	0.028 (3)	0.005 (2)	0.017 (2)	0.003 (2)
C2	0.028 (3)	0.047 (4)	0.039 (4)	−0.002 (3)	0.016 (3)	0.000 (3)
C3	0.060 (5)	0.034 (4)	0.058 (5)	−0.010 (3)	0.038 (4)	0.002 (3)
C4	0.040 (3)	0.022 (3)	0.038 (3)	0.007 (2)	0.017 (3)	0.011 (2)
C5	0.037 (4)	0.066 (5)	0.029 (3)	0.021 (3)	0.016 (3)	−0.001 (3)
N3	0.067 (4)	0.032 (3)	0.057 (4)	0.005 (3)	0.041 (4)	0.008 (3)
N4	0.055 (4)	0.025 (3)	0.037 (3)	−0.011 (2)	0.028 (3)	0.000 (2)
C6	0.032 (3)	0.024 (3)	0.034 (3)	0.001 (2)	0.019 (3)	0.001 (2)
C7	0.033 (3)	0.034 (3)	0.031 (3)	0.000 (2)	0.017 (3)	0.006 (2)
C8	0.033 (3)	0.027 (3)	0.052 (4)	−0.001 (3)	0.020 (3)	0.006 (3)
C9	0.030 (3)	0.023 (3)	0.028 (3)	−0.002 (2)	0.013 (2)	−0.007 (2)
C10	0.061 (5)	0.037 (4)	0.038 (4)	0.001 (3)	0.027 (4)	−0.009 (3)
N5	0.059 (4)	0.046 (4)	0.051 (4)	0.000 (3)	0.027 (4)	−0.005 (3)
N6	0.089 (5)	0.022 (3)	0.039 (3)	−0.005 (3)	0.021 (4)	0.006 (2)
C11	0.040 (3)	0.022 (3)	0.032 (3)	0.001 (2)	0.012 (3)	0.000 (2)
C12	0.062 (5)	0.028 (3)	0.035 (4)	0.008 (3)	0.013 (3)	−0.001 (3)
C13	0.058 (5)	0.024 (3)	0.052 (5)	0.003 (3)	0.010 (4)	0.001 (3)
C14	0.042 (4)	0.023 (3)	0.033 (3)	0.001 (3)	0.010 (3)	0.013 (2)
C15	0.049 (4)	0.046 (4)	0.035 (4)	0.004 (3)	0.013 (3)	0.012 (3)
N7	0.047 (4)	0.054 (4)	0.042 (3)	−0.007 (3)	0.023 (3)	0.000 (3)
N8	0.066 (4)	0.031 (3)	0.031 (3)	−0.014 (3)	0.015 (3)	−0.002 (2)
C16	0.034 (3)	0.029 (3)	0.032 (3)	0.000 (3)	0.013 (3)	0.001 (2)
C17	0.066 (5)	0.028 (3)	0.030 (3)	0.000 (3)	0.023 (3)	0.009 (3)
C18	0.057 (5)	0.040 (4)	0.028 (3)	0.006 (3)	0.011 (3)	−0.003 (3)
C19	0.031 (3)	0.025 (3)	0.034 (3)	−0.001 (2)	0.008 (3)	−0.011 (2)
C20	0.037 (4)	0.023 (3)	0.058 (5)	−0.006 (3)	0.021 (3)	−0.004 (3)
N9	0.050 (4)	0.053 (4)	0.092 (6)	−0.011 (3)	0.051 (4)	−0.005 (4)
N10	0.061 (4)	0.036 (3)	0.067 (5)	0.008 (3)	0.038 (4)	0.018 (3)
C21	0.030 (3)	0.033 (3)	0.031 (3)	−0.001 (2)	0.018 (2)	0.007 (2)
C22	0.030 (3)	0.044 (4)	0.056 (4)	−0.005 (3)	0.031 (3)	−0.004 (3)
C23	0.056 (4)	0.040 (4)	0.040 (4)	−0.021 (3)	0.031 (4)	−0.004 (3)
C24	0.036 (3)	0.016 (2)	0.033 (3)	0.004 (2)	0.012 (3)	0.005 (2)
C25	0.027 (3)	0.046 (4)	0.053 (4)	0.011 (3)	0.014 (3)	−0.001 (3)

### Pentakis(2-aminopyridinium) nonavanado(V)tellurate(VI) Geometric parameters (Å, º)

**Table:**

V1—O1E	1.595 (4)	Te1—O15	1.769 (4)
V1—O1	1.814 (4)	Te1—O18	1.774 (4)
V1—O2	1.867 (4)	Te1—O14	1.931 (4)
V1—O5	1.884 (4)	Te1—O13	1.950 (4)
V1—O15	2.067 (4)	Te1—O2C	2.052 (4)
V1—O1C	2.374 (4)	Te1—O1C	2.063 (4)
V2—O2E	1.601 (4)	Te2—O17	1.774 (4)
V2—O4	1.816 (4)	Te2—O16	1.789 (4)
V2—O2	1.818 (4)	Te2—O11	1.916 (4)
V2—O12	2.022 (4)	Te2—O12	1.923 (4)
V2—O14	2.039 (4)	Te2—O1C	2.048 (4)
V2—O1C	2.287 (4)	Te2—O2C	2.052 (4)
V3—O3E	1.611 (4)	N1—C5	1.347 (10)
V3—O1	1.824 (4)	N1—C1	1.409 (8)
V3—O4	1.848 (4)	N2—C1	1.307 (8)
V3—O6	1.901 (4)	C1—C2	1.420 (9)
V3—O16	2.041 (4)	C2—C3	1.343 (10)
V3—O1C	2.429 (4)	C3—C4	1.321 (10)
V4—O6	1.809 (4)	C4—C5	1.325 (11)
V4—O5	1.816 (4)	N3—C10	1.366 (10)
V4—O11	2.030 (4)	N3—C6	1.411 (9)
V4—O13	2.080 (4)	N4—C6	1.330 (8)
V4—O1C	2.285 (4)	C6—C7	1.410 (8)
V5—O5E	1.601 (4)	C7—C8	1.345 (10)
V5—O7	1.808 (4)	C8—C9	1.324 (9)
V5—O8	1.810 (4)	C9—C10	1.334 (10)
V5—O14	2.034 (4)	N5—C15	1.330 (10)
V5—O12	2.045 (4)	N5—C11	1.425 (9)
V5—O2C	2.277 (4)	N6—C11	1.322 (8)
V6—O6E	1.596 (4)	C11—C12	1.381 (9)
V6—O9	1.813 (4)	C12—C13	1.361 (10)
V6—O10	1.815 (4)	C13—C14	1.323 (11)
V6—O13	2.022 (4)	C14—C15	1.341 (10)
V6—O11	2.046 (4)	N7—C20	1.357 (9)
V6—O2C	2.296 (4)	N7—C16	1.444 (9)
V7—O7E	1.610 (4)	N8—C16	1.319 (8)
V7—O3	1.810 (4)	C16—C17	1.408 (9)
V7—O10	1.871 (4)	C17—C18	1.364 (10)
V7—O7	1.879 (4)	C18—C19	1.331 (10)
V7—O18	2.058 (4)	C19—C20	1.322 (10)
V7—O2C	2.396 (4)	N9—C25	1.379 (10)
V8—O8E	1.603 (4)	N9—C21	1.404 (9)
V8—O3	1.835 (4)	N10—C21	1.320 (8)
V8—O9	1.843 (4)	C21—C22	1.430 (9)
V8—O8	1.918 (4)	C22—C23	1.351 (10)
V8—O17	2.031 (4)	C23—C24	1.316 (10)
V8—O2C	2.414 (4)	C24—C25	1.309 (10)
			
O1E—V1—O1	104.6 (2)	O15—Te1—O13	95.61 (17)
O1E—V1—O2	104.1 (2)	O18—Te1—O13	95.62 (17)
O1—V1—O2	91.42 (19)	O14—Te1—O13	158.89 (16)
O1E—V1—O5	101.7 (2)	O15—Te1—O2C	166.18 (16)
O1—V1—O5	90.34 (19)	O18—Te1—O2C	88.46 (16)
O2—V1—O5	152.78 (17)	O14—Te1—O2C	81.97 (15)
O1E—V1—O15	99.6 (2)	O13—Te1—O2C	81.48 (15)
O1—V1—O15	155.70 (18)	O15—Te1—O1C	88.51 (16)
O2—V1—O15	84.15 (18)	O18—Te1—O1C	166.14 (16)
O5—V1—O15	83.16 (17)	O14—Te1—O1C	82.10 (15)
O1E—V1—O1C	173.4 (2)	O13—Te1—O1C	81.65 (15)
O1—V1—O1C	81.78 (16)	O2C—Te1—O1C	77.71 (14)
O2—V1—O1C	76.99 (15)	O17—Te2—O16	104.49 (18)
O5—V1—O1C	76.40 (15)	O17—Te2—O11	96.53 (17)
O15—V1—O1C	73.94 (14)	O16—Te2—O11	97.11 (17)
O2E—V2—O4	105.1 (2)	O17—Te2—O12	96.35 (17)
O2E—V2—O2	105.0 (2)	O16—Te2—O12	95.45 (17)
O4—V2—O2	93.47 (19)	O11—Te2—O12	159.15 (16)
O2E—V2—O12	99.83 (19)	O17—Te2—O1C	166.67 (16)
O4—V2—O12	90.20 (17)	O16—Te2—O1C	88.83 (17)
O2—V2—O12	152.94 (17)	O11—Te2—O1C	81.94 (15)
O2E—V2—O14	99.1 (2)	O12—Te2—O1C	81.75 (15)
O4—V2—O14	153.70 (17)	O17—Te2—O2C	88.63 (16)
O2—V2—O14	90.08 (17)	O16—Te2—O2C	166.84 (17)
O12—V2—O14	75.45 (15)	O11—Te2—O2C	82.04 (14)
O2E—V2—O1C	171.85 (19)	O12—Te2—O2C	81.94 (15)
O4—V2—O1C	80.48 (16)	O1C—Te2—O2C	78.04 (14)
O2—V2—O1C	80.23 (16)	Te2—O1C—Te1	102.01 (15)
O12—V2—O1C	73.96 (14)	Te2—O1C—V4	94.00 (14)
O14—V2—O1C	74.46 (14)	Te1—O1C—V4	95.66 (14)
O3E—V3—O1	105.6 (2)	Te2—O1C—V2	94.16 (14)
O3E—V3—O4	104.6 (2)	Te1—O1C—V2	94.01 (15)
O1—V3—O4	90.89 (19)	V4—O1C—V2	165.78 (17)
O3E—V3—O6	103.5 (2)	Te2—O1C—V1	168.96 (19)
O1—V3—O6	89.30 (19)	Te1—O1C—V1	89.03 (13)
O4—V3—O6	150.70 (17)	V4—O1C—V1	85.12 (12)
O3E—V3—O16	100.9 (2)	V2—O1C—V1	84.64 (12)
O1—V3—O16	153.48 (17)	Te2—O1C—V3	88.27 (13)
O4—V3—O16	84.20 (17)	Te1—O1C—V3	169.63 (18)
O6—V3—O16	82.68 (17)	V4—O1C—V3	84.84 (12)
O3E—V3—O1C	174.3 (2)	V2—O1C—V3	83.79 (12)
O1—V3—O1C	80.04 (15)	V1—O1C—V3	80.69 (11)
O4—V3—O1C	76.06 (15)	Te2—O2C—Te1	102.24 (15)
O6—V3—O1C	75.12 (15)	Te2—O2C—V5	94.46 (14)
O16—V3—O1C	73.48 (14)	Te1—O2C—V5	94.28 (14)
O4E—V4—O6	105.1 (2)	Te2—O2C—V6	94.38 (14)
O4E—V4—O5	104.1 (2)	Te1—O2C—V6	94.46 (13)
O6—V4—O5	95.27 (19)	V5—O2C—V6	165.98 (17)
O4E—V4—O11	100.81 (19)	Te2—O2C—V7	168.78 (18)
O6—V4—O11	89.92 (17)	Te1—O2C—V7	88.98 (13)
O5—V4—O11	152.19 (17)	V5—O2C—V7	84.49 (12)
O4E—V4—O13	99.75 (19)	V6—O2C—V7	84.73 (12)
O6—V4—O13	152.93 (17)	Te2—O2C—V8	88.04 (13)
O5—V4—O13	88.79 (16)	Te1—O2C—V8	169.69 (18)
O11—V4—O13	74.93 (15)	V5—O2C—V8	85.54 (12)
O4E—V4—O1C	172.39 (18)	V6—O2C—V8	83.91 (12)
O6—V4—O1C	80.60 (16)	V7—O2C—V8	80.74 (11)
O5—V4—O1C	80.03 (15)	V1—O1—V3	117.4 (2)
O11—V4—O1C	73.89 (14)	V2—O2—V1	116.8 (2)
O13—V4—O1C	73.76 (14)	V7—O3—V8	117.5 (2)
O5E—V5—O7	104.3 (2)	V2—O4—V3	118.6 (2)
O5E—V5—O8	104.1 (2)	V4—O5—V1	116.8 (2)
O7—V5—O8	95.22 (18)	V4—O6—V3	118.0 (2)
O5E—V5—O14	99.98 (19)	V5—O7—V7	116.9 (2)
O7—V5—O14	89.63 (17)	V5—O8—V8	117.5 (2)
O8—V5—O14	153.42 (17)	V6—O9—V8	119.0 (2)
O5E—V5—O12	100.09 (19)	V6—O10—V7	118.1 (2)
O7—V5—O12	153.11 (17)	Te2—O11—V4	107.03 (17)
O8—V5—O12	89.60 (16)	Te2—O11—V6	107.39 (17)
O14—V5—O12	75.06 (15)	V4—O11—V6	101.05 (16)
O5E—V5—O2C	172.62 (18)	Te2—O12—V2	107.36 (17)
O7—V5—O2C	80.70 (15)	Te2—O12—V5	106.61 (17)
O8—V5—O2C	80.62 (16)	V2—O12—V5	101.10 (17)
O14—V5—O2C	74.39 (14)	Te1—O13—V6	107.13 (18)
O12—V5—O2C	74.01 (14)	Te1—O13—V4	106.28 (16)
O6E—V6—O9	105.6 (2)	V6—O13—V4	100.15 (15)
O6E—V6—O10	104.8 (2)	Te1—O14—V5	106.48 (17)
O9—V6—O10	93.46 (18)	Te1—O14—V2	106.64 (17)
O6E—V6—O13	99.6 (2)	V5—O14—V2	100.86 (17)
O9—V6—O13	152.42 (17)	Te1—O15—V1	108.51 (19)
O10—V6—O13	90.62 (16)	Te2—O16—V3	109.4 (2)
O6E—V6—O11	101.0 (2)	Te2—O17—V8	109.70 (19)
O9—V6—O11	88.47 (17)	Te1—O18—V7	109.11 (19)
O10—V6—O11	152.55 (17)	C5—N1—C1	119.7 (6)
O13—V6—O11	75.85 (15)	N2—C1—N1	122.8 (6)
O6E—V6—O2C	172.43 (19)	N2—C1—C2	121.3 (6)
O9—V6—O2C	79.70 (16)	N1—C1—C2	115.9 (6)
O10—V6—O2C	79.87 (16)	C3—C2—C1	120.3 (7)
O13—V6—O2C	74.19 (14)	C4—C3—C2	121.4 (7)
O11—V6—O2C	73.54 (14)	C3—C4—C5	120.7 (6)
O7E—V7—O3	104.7 (2)	C4—C5—N1	122.1 (7)
O7E—V7—O10	104.1 (2)	C10—N3—C6	120.7 (6)
O3—V7—O10	90.59 (18)	N4—C6—C7	121.6 (6)
O7E—V7—O7	102.8 (2)	N4—C6—N3	122.3 (6)
O3—V7—O7	90.85 (18)	C7—C6—N3	116.1 (6)
O10—V7—O7	151.82 (17)	C8—C7—C6	119.6 (6)
O7E—V7—O18	100.5 (2)	C9—C8—C7	122.6 (6)
O3—V7—O18	154.80 (17)	C8—C9—C10	120.7 (6)
O10—V7—O18	83.70 (17)	C9—C10—N3	120.2 (7)
O7—V7—O18	83.10 (17)	C15—N5—C11	120.3 (7)
O7E—V7—O2C	173.89 (19)	N6—C11—C12	122.2 (6)
O3—V7—O2C	81.35 (15)	N6—C11—N5	121.1 (6)
O10—V7—O2C	76.18 (15)	C12—C11—N5	116.6 (6)
O7—V7—O2C	76.23 (15)	C13—C12—C11	120.0 (7)
O18—V7—O2C	73.45 (14)	C14—C13—C12	121.4 (7)
O8E—V8—O3	105.8 (2)	C13—C14—C15	120.6 (6)
O8E—V8—O9	103.5 (2)	N5—C15—C14	121.1 (7)
O3—V8—O9	91.99 (18)	C20—N7—C16	119.0 (6)
O8E—V8—O8	104.8 (2)	N8—C16—C17	122.6 (6)
O3—V8—O8	88.41 (18)	N8—C16—N7	120.6 (6)
O9—V8—O8	150.53 (17)	C17—C16—N7	116.8 (6)
O8E—V8—O17	100.3 (2)	C18—C17—C16	119.2 (6)
O3—V8—O17	153.80 (17)	C19—C18—C17	122.1 (7)
O9—V8—O17	84.52 (17)	C20—C19—C18	121.1 (6)
O8—V8—O17	82.30 (16)	C19—C20—N7	121.9 (7)
O8E—V8—O2C	173.9 (2)	C25—N9—C21	119.3 (6)
O3—V8—O2C	80.36 (15)	N10—C21—N9	122.3 (6)
O9—V8—O2C	75.98 (14)	N10—C21—C22	121.9 (6)
O8—V8—O2C	75.05 (14)	N9—C21—C22	115.8 (6)
O17—V8—O2C	73.59 (14)	C23—C22—C21	119.5 (6)
O15—Te1—O18	105.31 (18)	C24—C23—C22	122.7 (6)
O15—Te1—O14	97.35 (17)	C25—C24—C23	120.0 (6)
O18—Te1—O14	96.93 (18)	C24—C25—N9	122.5 (6)

### Pentakis(2-aminopyridinium) nonavanado(V)tellurate(VI) Hydrogen-bond geometry (Å, º)

**Table:**

*D*—H···*A*	*D*—H	H···*A*	*D*···*A*	*D*—H···*A*
N1—H1···O6^i^	0.86	2.57	3.426 (8)	172
N2—H2*A*···O1^i^	0.86	2.26	3.105 (8)	167
N2—H2*B*···O3^ii^	0.86	2.50	3.006 (7)	118
N2—H2*B*···O10^ii^	0.86	2.28	3.121 (8)	165
C4—H4···O3*E*	0.93	2.44	2.995 (8)	119
C4—H4···O8*E*^iii^	0.93	2.26	3.073 (8)	146
C5—H5···O9^iii^	0.93	2.24	3.136 (8)	161
N3—H3*A*···O12^iv^	0.86	2.54	3.397 (7)	175
N4—H4*A*···O7^v^	0.86	2.05	2.896 (7)	168
N4—H4*B*···O16^iv^	0.86	2.19	2.998 (7)	156
C7—H7···O18^v^	0.93	2.57	3.495 (8)	171
C9—H9···O5^i^	0.93	1.86	2.760 (7)	161
N5—H5*A*···O10^ii^	0.86	2.61	3.404 (8)	154
N6—H6*A*···O1*E*^vi^	0.86	2.14	2.922 (8)	151
N6—H6*B*···O7*E*^ii^	0.86	2.05	2.900 (8)	171
C12—H12···O1*E*^vi^	0.93	2.63	3.312 (9)	130
C14—H14···O8^iv^	0.93	1.78	2.703 (7)	174
C14—H14···O17^iv^	0.93	2.62	3.146 (8)	117
N7—H7*A*···O15^vi^	0.86	2.48	3.308 (8)	161
N8—H8*A*···O3*E*^vii^	0.86	2.12	2.966 (7)	169
N8—H8*B*···O14^vi^	0.86	2.18	2.963 (7)	152
C19—H19···O13^ii^	0.93	1.88	2.788 (7)	165
C20—H20···O18^ii^	0.93	2.43	3.202 (8)	140
N9—H9*A*···O5*E*	0.86	2.46	3.244 (7)	151
N10—H10*A*···O3	0.86	2.30	3.098 (8)	155
N10—H10*B*···O1^viii^	0.86	2.65	3.294 (8)	132
N10—H10*B*···O2^viii^	0.86	2.34	3.169 (8)	162
C22—H22···O2*E*^viii^	0.93	2.29	3.189 (8)	164
C24—H24···O4*E*^ix^	0.93	2.35	2.929 (7)	120
C24—H24···O6*E*^ix^	0.93	2.61	3.153 (7)	118
C24—H24···O11^x^	0.93	2.48	3.003 (7)	116
C10—H10···*Cg*1	0.93	3.07	3.902	151
C18—H18···*Cg*5^iv^	0.93	2.66	3.450	143

Symmetry codes: (i) −*x*, −*y*, −*z*+1; (ii) −*x*+1, −*y*, −*z*+1; (iii) *x*−1, *y*, *z*; (iv) *x*, −*y*+1/2, *z*+1/2; (v) *x*, *y*, *z*+1; (vi) *x*+1, *y*, *z*+1; (vii) *x*+1, −*y*+1/2, *z*+1/2; (viii) *x*+1, *y*, *z*; (ix) −*x*+1, *y*+1/2, −*z*+1/2; (x) *x*, −*y*+1/2, *z*−1/2.
